# The survival and dispersal of *Taenia* eggs in the environment: what are the implications for transmission? A systematic review

**DOI:** 10.1186/s13071-021-04589-6

**Published:** 2021-01-29

**Authors:** Famke Jansen, Pierre Dorny, Sarah Gabriël, Veronique Dermauw, Maria Vang Johansen, Chiara Trevisan

**Affiliations:** 1 Department of Biomedical Sciences, Institute of Tropical Medicine, 155 Nationalestraat, 2000 Antwerp, Belgium; 2 Department of Veterinary Public Health and Food Safety, Faculty of Veterinary Medicine, Ghent University, 133 Salisburylaan, 9820 Merelbeke, Belgium; 3 Department of Veterinary and Animal Sciences, Faculty of Health and Medical Sciences, University of Copenhagen, 100 Dyrlægevej, 1870 Frederiksberg, Denmark

**Keywords:** *Taenia*, Egg survival, Spread, Environment, Sewage treatment

## Abstract

*Taenia* spp. are responsible for a substantial health and economic burden in affected populations. Knowledge of the fate of the eggs of *Taenia* spp. in the environment and of other factors facilitating the transmission of eggs to intermediate hosts is important for the control/elimination of infections caused by *Taenia* spp. The aim of this systematic review was to summarize current knowledge of the factors influencing the survival and dispersal of *Taenia* spp. eggs in the environment. Publications retrieved from international databases were systematically reviewed. Of the 1465 papers initially identified, data were ultimately extracted from 93 papers. The results of this systematic review indicate that survival is favoured at moderate temperatures (0–20 °C). Humidity seems to affect the survival of *Taenia* spp. eggs more than temperature. Under field circumstances, *Taenia* spp. eggs have been found to survive for up to 1 year.* Taenia* spp. eggs are commonly found on vegetables (0.9–30%) and in soil and water samples (0–43%), with their presence posing a risk to the consumer. Invertebrates may act as transport hosts, transferring the infection to an intermediate host, but the importance of this route of transmission is still open to question. Wastewater treatment systems are not capable of entirely eliminating *Taenia* spp. eggs. Access to surface water and the use of sewage sludge as fertilizer on pastures are important risk factors for bovine cysticercosis. Although information on the survival and spread of *Taenia* spp. eggs is available, in general the data retrieved and reviewed in this article were old, focused on very specific geographical regions and may not be relevant for other areas or not specific for different *Taenia* spp. Furthermore, it is unknown whether egg survival differs according to *Taenia* sp. Future studies are necessary to identify sustainable methods to identify and inactivate parasite eggs in the environment and reduce their spread. 
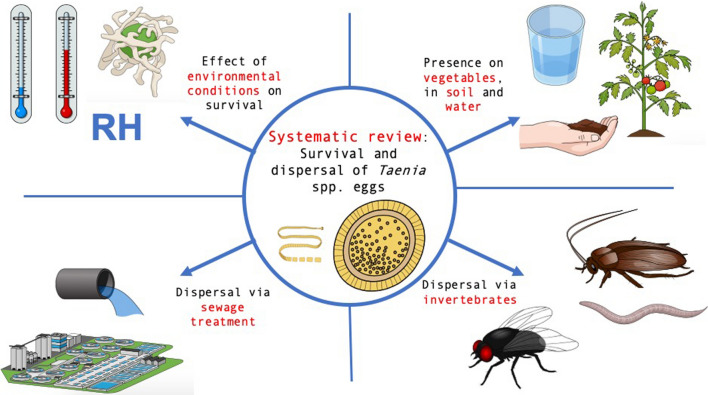

## Background

*Taenia* spp. are important tapeworm species in humans and domesticated animals that may lead to a substantial health and economic burden [1–3]. Humans are the sole definitive hosts of three zoonotic *Taenia* spp., namely *T. saginata*, *T. solium* and *T. asiatica* [4]. Other *Taenia* spp., such as *T. hydatigena*,* T. pisiformis*,* T. ovis*,* T. taeniaeformis* and *T. multiceps*, are mainly of veterinary importance. *Taenia saginata* is the most common and most widely distributed tapeworm in the human host [5]. *Taenia solium*, on the other hand, is endemic in large parts of Asia, Latin America and sub-Saharan Africa, while *T. asiatica* seems to be restricted to Asia [3]. Infections with *T. solium* and *T. asiatica* are considered to be neglected tropical diseases, and especially for infections caused by the former, the call for control and elimination is warranted as the parasite can also cause cysticercosis in humans. The establishment of cysticerci in the central nervous system may lead to neurocysticercosis, which has been found to be associated with more than 30% of acquired epilepsy cases in endemic regions [6–9].

Humans become infected with *T. saginata*,* T. solium* and *T. asiatica* by consuming raw or undercooked infected beef, pork or pig organs containing cysticerci, the metacestode larvae of the tapeworm. Upon ingestion of a viable cysticercus, an adult tapeworm may develop that resides in the intestinal lumen of the human final host [3, 6]. Infection with a tapeworm (taeniosis) generally remains asymptomatic [10, 11] with some exceptions [12–14].

Gravid proglottids containing infective eggs are shed with the stool of the definitive host; in the case of *T. saginata* they may also be expelled independently of defecation [3]. In industrialized countries, inadequately treated sewage is generally considered to contribute to infections in cattle by *T. saginata*, as animals become infected by ingesting the eggs from contaminated pastures after flooding or from access to surface water [11]. On the other hand, in low-income countries, humans contaminate the environment (soil, crops and water) with *Taenia* spp. eggs present in faeces due to poor hygienic standards and the lack of latrines [15]. In general, contamination of food, soil and water can increase the risk of infection for humans (*T. solium*) and other intermediate hosts (all *Taenia* spp.) [16–18], as does possible spread* via* invertebrates and wind [19, 20].

Control and treatment options for *Taenia* spp. have generally been generated from a two-compartment approach, with the focus either on the definitive host or on the intermediate host. Interventions for *T. solium*, including education, meat inspection, sanitation, treatment of final and intermediate hosts and pig vaccination, have been implemented, either as single interventions or in combination [21]. However, focus on the third compartment, namely the egg stage in the environment, has often been neglected even though tapeworms have the ability to produce up to 300,000 eggs each day [22]. Therefore, egg survival and dispersal studies can lead to new insights on the survival capacity of eggs and to possible new control options to break the life-cycle of these parasites and prevent infection of cattle, pigs and humans. In general, egg survival experiments are conducted under *in vivo* or *in vitro* conditions. In *in vitro* experiments, eggs are checked for viability based on integrity (mostly morphological determination), hatching and activation (movement of the larva after hatching), the latter two approaches performed in designated media mimicking gastric juices [23–25]. These terms are often used interchangeably, so caution is necessary when interpreting study findings. In *in vivo* studies, egg infectivity is determined by feeding eggs to naïve intermediate host animals followed by dissecting the carcasses for cysticerci recovery [26].

The aim of the systematic review was to review current knowledge of the factors that influence the survival and dispersal of *Taenia* spp. eggs in the environment. More specifically, we aimed to summarize current knowledge on (i) the survival of *Taenia* spp. eggs under specific temperature and relative humidity (RH) conditions in laboratory and field experiments; (ii) the presence of eggs on vegetables, fruit, soil and water depending on the geographical area or climate zone of the study; (iii) the spread of eggs via different means, such as invertebrates and wind; and finally, (iv) the importance of sewage treatment systems in egg dispersal.

## Methods

A systematic review of literature published up to 31 July 2019 was conducted to collect information on the survival and dispersal of *Taenia* spp. eggs in the environment, using an approach that followed PRISMA guidelines [27]. No restriction was made on publication date. The protocol and the PRISMA checklist for this review can be found in Additional file 1 and Additional file 2, respectively. Two search engines, PubMed (http://www.ncbi.nlm.nih.gov/pubmed) and Web of Science (www.webofknowledge.com), were searched without the use of a specific time frame and using the following keywords and Boolean opeators: taeni* AND egg* AND (surviv* OR viab* OR resist* OR longevi* OR activ* OR hatch* OR transmi* OR epi* OR infectiv* OR water OR wastewater OR sewage OR sludge OR river OR stream OR soil OR silt OR grass OR saline OR environment* OR medi*).

Outputs from the two search engines were first screened for the English language, and publications in languages other than English were excluded. The results were then compiled and screened for duplicates, after which titles and abstracts were screened for eligibility by two independent reviewers. Publications were excluded based on the following reasons: (i) studies on species other than *Taenia* spp.; (ii) studies outside the scope of this review (egg survival and dispersal), such as laboratory techniques for hatching; and (iii) reviews and editorial letters. Where possible, full texts were retrieved and evaluated according to the same criteria. The reference lists of each eligible article were also screened for relevant literature. Data were extracted from the records into predefined tables using Microsoft Excel (Microsoft Corp., Redmond, WA, USA).

## Results

**Fig. 1:**
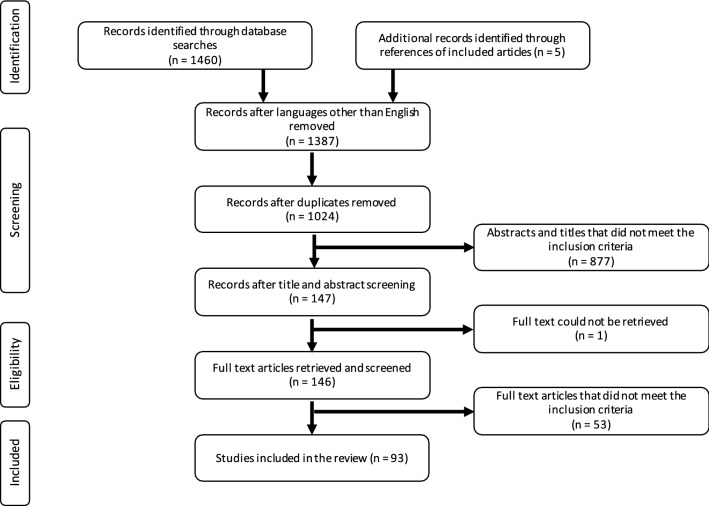
Flow diagram of the database search

A total of 1460 publications were identified through the database searches, and an additional five articles were identified after screening the relevant literature. Ninety-three studies were included in the systematic review after careful elimination of the remaining papers based on the exclusion criteria (Fig. 1).

### Egg survival

Twenty-four studies were identified that investigated *Taenia* spp. egg survival in the environment. The studies shown in Table 1 describe laboratory or field experiments aimed at determining the survival of eggs after exposure to a range of temperatures and relative humidities, to different light types and to various media. In general, humidity seems to affect *Taenia* spp. egg survival more than temperature, with low humidity (< 34%) hampering survival. Moderate temperatures (between 5 °C and 25 °C) favour survival, while warmer temperatures (> 25 °C) and freezing shorten survival times. Under field conditions, survival is dependent on the specific *Taenia* sp. studied and the specific outdoor conditions. In one study on Kenyan pastures, eggs were observed to survive up to 1 year [28].

**Table 1:** Summary of available literature on *Taenia* spp. egg survival capacity

Test conditions	Species	Egg viability test	Result	Year of publication	Reference
− 4 °C for 4.5, 12.3 and 76.1 days	*T. saginata*	*In vivo*: 300,000 eggs fed to one calf per condition	Heavy infection after 4.5 days, moderate after 12.3 days and only 2 cysticerci after 76.1 days	1960	[77]
Between − 9 °C and 38 °C	*T. hydatigena* and *T. ovis*	*In vivo*: feeding to 8 lambs on days 90 and 273 (2000 *T. hydatigena* eggs each (eggs stored at − 9 °C and 7 °C fed to lambs on days 90 and 273).	Cysticerci formed after 90 days (279 at 7 °C and 14 at −9 °C) and after 273 days at 7 °C (6 cysticerci), but not at − 9°C	1977	[74]
4 °C in saline for 28 and 91 days	*T. taeniaeformis*	*In vivo*: intragastric application to mice	36% cyst recovery after 28 days and 10% after 91 days	1990	[68]
5-min-long heat treatment at 50 °C or 60°C, with 22 °C as control	*T. hydatigena*	*In vivo*: 100–2000 eggs fed to lambs and cysticerci counted	The mean percentage of cysticerci recovered were 11.55, 1.22 and 0 % for 22 °C, 50 °C and 60°C, respectively	2013	[95]
4–5 °C in 1:10,000 merthiolate in normal saline	*T. saginata*	*In vitro* activation (Silverman’s hatching technique) and *in vivo* (calves)	Hatching (*in vitro*) and infectivity/survival (*in vivo*) for at least 168 days	1962	[73]
Between − 9 °C and 38 °C	*T. hydatigena* and *T. ovis*	*In vitro* hatching and activity: AGF and AIF	*In vitro*: Temperature-dependent decline in embryo activity	1977	[74]
At 4 °C and at − 20 °C, − 9 °C, − 4 °C, 0 °C, 10 °C, 18–22 °C, 25 °C and 37 °C in saline with antibiotics and mycostatin (for up to 42 days)	*T. multiceps*	*In vitro* hatching, activation	4°C: Activation levels increased up to 27 days (55%) but decreased afterwards (22% after 50 days). Eggs withstand freezing at − 20 °C for 42 days. Hatching ability lost after 7 days at 37 °C. Intermediate temperature shows no or negligible effects on activation	1984	[75]
5-min-long heat treatment at 40 °C, 45 °C, 50 °C, 55 °C or 60 °C; 22 °C as control	*T. hydatigena*	*In vitro:* hatching	*In vitro*: Activation of 6.14% of 3.07% recovered eggs after treatment at 40 °C. 99.47% reduction in activation or infectivity after treatment at 60 °C	2013	[95]
5 °C and 20 °C in water and in silt for 2, 4 and 6 months and − 18°C for 1 week	*T. saginata*	*In vitro*: hatching with NaClO and activation with AIF	Activation after 6 months at 5 °C. Decreased activation at 20 °C: 4 months in water and 2 months in silt. Activation after 1 week at − 18°C	2019	[78]
Desiccation (on glass slides until water is evaporated), mild temperature (38 °C)	*T. pisiformis, T. ovis, T. hydatigena, Echinococcus granulosus*	*In vitro*: treatment with AIF or AGF (integrity, hatching and activation)	Desiccation most restrictive for survival, *T. pisiformis* and *E. granulosus* more susceptible	1968	[72]
RH of 31, 47 and 89% (19.5 °C) for up to 365 dats	*T. hydatigena*	*In vitro*: vitality = ability to exclude 0.1% aqueous trypan blue	Vitality: 80–82% at 89% RH, 59.5–64.5% at 47% RH and 38–40% at 31% RH. Reduced vitality: 93.4% at 31% RH, 93.15% at 47% RH and 73.58% at 89% RH	2017	[96]
High T (37 - 39°C) or low T (3 - 5°C) AND high RH (89-94%) or low RH (32-33%)	*T. pisiformis*	*In vivo*: 2000 eggs fed to 7–10 rabbits in each group (control group received fresh sample of eggs at the beginning of the experiment)	High cyst count in control group (193 cysticerci). High RH: Average 3 cysticerci after 300 days (low temperature) and 0.5 after 7 days (high temperature). Low RH: 21 cysticerci after 56 days (low temperature) and no recovery (high temperature)	1975	[76]
Between 7 °C and 65 °C plus desiccation (water removed) (up to 300 days)	*T. hydatigena, T. ovis* and *T, multiceps*	*In vitro* hatching: AGF and AIF	No hatching after 4 days at 38 °C and higher (*T. ovis* and *T. hydatigena*). Hatching after 56 days at 21 °C and 294 days at 7°C	1977	[74]
High temperature (30–80 °C), different RH (10, 80, 90 and 95%) and different contact times	*T. solium* (and other helminth genera)	*In vitro* hatching with NaClO solution	For complete inactivation of *T. solium* eggs: combination of temperature > 70C and 80% RH for 120 min	2010	[97]
Pastures contaminated with egg suspensions (2000–4000 eggs/sq. yard): high rainfall *vs* dry (Kenya)	*T. saginata*	*In vivo*: 14 calves allowed to graze for 3 to 143 days (13–413 days after infection pasture)	Cysticerci found in 12 calves, for both pasture types. Highest number of recovered cysticerci was 233 in calf allowed to graze for 142 days, start of grazing 304 days after infection pasture (3400 eggs/ sq.yard)	1948	[28]
Outdoor on patch of 2 0m^2^ (Australia): winter (0–20 °C) and summer (8–30 °C)	*T. pisiformis*	*In vivo*: rabbits grazing on patch (maximum 126 days)	Winter: Gradual decrease of infectivity over time, recovery after 126 days. Summer: No infection after 14 days	1975	[76]
Batches of 11,500 eggs deposited outdoors on natural soil surface (starting from May and September for up to 9.5 months)	*T. saginata*	*In vivo*: batches fed to calves	Deposited in May: Infective for 6.5 months, not for 9.5 months. Deposited in September: Infective for 5.5 months, not for 8.5 months	1990	[80]
Outdoor storage (May–June) in UK	*T. multiceps*	*In vitro* hatching, activation	Hatching ability lost after 28 days	1984	[75]
In freshwater stream in Denmark from December to February (fluctuating temperature: − 10 to 17 °C) and outdoors for 1 week in February (− 6-5 °C)	*T. saginata*	*In vitro*: hatching with NaClO and activation with AIF	Activation after 4 months in stream AND repeated freezing, thawing outdoors 1 week	2019	[78]
Ensilation of eggs with minced potato (up to 28 days)	*T. hydatigena*	*In vivo*: 2000 eggs fed to lambs and cysticerci counted	359 cysticerci after 0 days, 5 cysticerci after 2 days, no cysticerci after 28 days. Reduction of 99.9% after 18.59 days of ensilation	2013	[98]
Gravid segments fixed in 70% EtOH or FA or frozen for 1 week	*T. taeniaeformis*	*In vitro* hatching (0.5% NaClO method)	No hatching of eggs from formalized segments. Hatching of eggs in all other conditions: eggs fragile after freezing; highest hatching rates after fixation in EtOH (12.6–69.2%)	1994	[99]
Exposure to UV radiation (eggs and eggs freed of the embryophore): 254 nm, dose rate of 0.6 mJ/cm^2^/s for 600 to 9600 s or 60 mJ/cm^2^/s for 6 to 192 s	*T. taeniaeformis*	*In* vivo: oral inoculation of 5000 eggs in rats	Number of cysticerci decreased dose dependently and no cysticerci were recovered after exposure to a total dose of 2880 mJ/cm^2^. After removal of embryophore, no cysticercus development after a total dose of 30 mJ/m^2^	1997	[100]
Exposure to UV radiation: UVA (near UV wavelength of 320–400 nm), UVB (mid UV, 290–320 nm) and UVC (far UV, 200–290 nm) for 30, 90, 270, 810, 2430 and 7290 s	*T. taeniaeformis*	*In vivo*: oral inoculation of 3,000 eggs in rats	UVC exposure significant effect on number of cysticerci from 90 s of exposure onwards (and smaller cysts), 100% reduction from 2430 s of exposure onwards. UVA reduction was 31.9 and 28.3% and UVB reduction was 51.8 and 54.8% at 2430 and 7290 s, respectively	2001	[101]
Sunlight (0 °C; 24 h) and UV light (20 °C; 24 h, 8 days)	*T. pisiformis*	*In vitro*: treatment with AIF or AGF (integrity, hatching and activation)	No difference in integrity, hatching and activation between control and treated eggs	1968	[72]
Exposure to UV radiation (250 nm), up to 48h	*T. multiceps*	*In vitro* hatching, activation	Hatching reduced to 3% after 24 h	1984	[75]
Lime dose of 15 and 20% CaO w/v dry basis with 80 or 90% humidity	*T. solium* (and other helminth genera)	*In vitro:* hatching with NaClO solution	For complete inactivation: 20% CaO dose (pH 12.5) and 80% humidity for 5 months	2010	[97]
High temperature (25–80 °C), different RH (10, 80, 90 and 95% RH), pH (neutral, 15 – 20% quicklime), contact times	*T. solium* (and other helminth genera)	*In vitro:* hatching with NaClO solution	For complete inactivation: combination of temperature > 70 °C and 80% humidity for 120 min OR pH 5.3, 45 °C, 90% RH for 6 days OR pH 12.7, 45 °C, 90% RH for 19 days	2012	[102]

Studies are ordered by test conditions (temperature, RH, season, UV radiation, combination of conditions) and within this order by *in vivo* vs. *in vitro* studies, and then by publication year (oldest to most recent).* In vitro* studies focus on integrity (morphological), hatching and activation (movement of the larvae), and* in vivo* studies focus on infectivity and survival determined by recovery of cysticerci

AGF, Artificial gastric fluid; AIF, artificial intestinal fluid; CaO, calcium oxide; EtOH, ethanol; FA, formaldehyde; NaClO, sodium hypochlorite; RH, relative humidity; UV ultraviolet

Studies investigating the effect of heat treatment (> 40°C) were generally not directed at environmental factors affecting survival but more focussed on which factors were effective in destroying eggs (in this case, cooking or boiling of food and fluids). The ovicidal activity of several naturally occurring agents was investigated. A number of studies reported that the fungi *Paecolimyces lilacinus* and *Pochonia chlamydosporia* were able to colonize the egg contents of *T. saginata* and *T. taeniaeformis* eggs, which led to their destruction [29–33]. It was also reported that lime nitrogen had the most destructive effect on egg survival of all fertilizers tested, with the eggs only surviving for 2 days in this substance; survival in other fertilizers was 2 days in limestone, 10 days in ammonium nitrate with limestone, 3 days in superphosphate substance, 3–7 days in NPK3, 10 days in potash salt and 30–35 days in urea [34].

### Environmental spread of eggs

A total of 43 papers, representing the majority of all publications retained in this review, described possible means of spreading of *Taenia* spp. eggs in the environment. Fifteen papers investigated the presence of helminth eggs on vegetables bought at markets, and a number also examined the effect of washing of vegetables on the number of eggs (Table 2). In general, prevalence of *Taenia* spp. eggs found on fruits and vegetables is high, ranging from 0.9 to 33%.

**Table 2:** Overview of results on *Taenia* spp. egg prevalence on vegetables and fruit

Country	Climate zone^a^	*Taenia* spp. egg prevalence before washing, no species specified (*N* = sample size)	*Taenia* spp. egg prevalence after washing (*N* = sample size)	Year of publication	Reference
Iran	Hot desert with Mediterranean and continental hot summer climate in the north	0.9% (*N *= 772)	0 (*N *= 772)	2016	[41]
Iran	Hot desert with Mediterranean and continental hot summer climate in the north	9.2% (*N* = 304)	1.3% (*N* = 304) (traditional washing^b^) 0 (standard washing^c^)	2012	[45]
Iran	Hot desert with Mediterranean and continental hot summer climate in the north	1.8% *(N* = 218) (*Taenia/Echinococcus*)	0 (*N* = 436)	2010	[42]
Iran	Hot desert with Mediterranean and continental hot summer climate in the north	4.86% (*N* = 453)	NA^d^	2016	[37]
Nigeria	Tropical Savanna climate with hot semi-arid climate in the north	10.6% (*N* = 199)	NA	2012	[40]
Nigeria	Tropical savanna climate with hot semi-arid climate in the north	1.25% (*N* = 960)	NA	2015	[36]
Nigeria	Tropical savanna climate with hot semi-arid climate in the north	2% (*N* = 1130) (*Taenia/Echinococcus*)	NA	2012	[35]
Pakistan	Hot desert with hot semi-arid climate in the north	2.7% (*N* = 520)	NA	2017	[39]
Turkey	Mixed cold semi-arid, Mediterranean and continental hot summer climate	3.5% (*N* = 203)	0 (*N* = 406)	2005	[84]
Turkey	Mixed cold semi-arid, Mediterranean and continental hot summer climate	2.7% (*N* = 111) (*Taenia/Echinococcus*)	NA	2013	[103]
Jordan	Hot desert	6% (*N* = 133)	NA	2016	[38]
Saudi Arabia	Hot desert	3.2% (*N* = 470) (*Taenia/Echinococcus*)	NA	2010	[44]
Vietnam	Tropical savanna climate	<1% (*N* = 317)	NA	2009	[43]
Libya	Hot desert	*Taenia/Echinococcus* spp.: in 6% of tomato (*N* = 36), 25% of cucumber (*N* = 36), 33% of lettuce (*N* = 27) and 30% of cress samples (*N *= 27)	NA	2010	[83]

^a^Köppen climate classification

^b^Immersed in tap water in sink for 6-7 min, then, rinsed for 1.5–2 mins

^c^Washed, immersed in solution containing 200 ppm active calcium hypochlorite for 30 mins, rinsed in automated fruit-vegetable washer.

^d^Not applicable, not investigated in the study

Research on the contamination of fruits and vegetables has been conducted in only few countries and consequently in only a few climate zones. Although all five major climate zones are represented in the studies reviewed, many of the climate subdivisions are not. Survival of eggs was found to be very dependent on temperature and RH and, therefore, also on climate zone. The authors of most studies agreed that leafy vegetables had a higher prevelance of parasites than smooth vegetables, such as tomatoes and cucumbers [35–43]. Parasite egg prevalence in general, and the prevalence of *Taenia* spp. eggs specifically, was higher in the summer and spring compared to the winter and autumn [37, 44, 45].

Federer et al. [17] studied the presence of taeniid DNA by multiplex-PCR in the water used to wash the fruit and vegetable mixes fed to zoo animals in Switzerland. The vegetables and fruits in the mix originated from all over Europe. In the autumn, 18% of the water samples contained taeniid DNA, compared to 28% in the spring.

Eleven papers reported on egg presence in soil and water samples (Table 3). Again, most articles focussed on all parasitic material found, and the results for *Taenia* spp. eggs were only a small part of the total results. In general, prevalence ranged from 0 to 43%.

**Table 3:** Overview of results on *Taenia* spp. egg presence in soil and water and on objects

Country	Climate zone^a^	Medium	Identification method	Result (*N* = sample size)	Year of publication	Reference
Cameroon	Tropical monsoon and tropical savanna climate	Water: Marshy areas	Formalin–ether concentration and Kato-Katz technique	*Taenia* spp. eggs detected with a maximum of 118 eggs/l in short rainy season (*N* = 96)	2019	[109]
Canada	Subarctic and tundra climate	Sediment: Water supply for cattle	Sedimentation and Sheather’s flotation technique	9 eggs in total (*N* = 482)	2004	[86]
Iraq	Hot desert, hot semi-arid and Mediterranean hot summer climate	Soil: Public squares and parks	Zinc sulphate flotation	6.2% (*Taenia* spp.) (*N* = 48)	2015	[104]
Mexico	Tropical, arid and semi-arid climate	Soil: In village with porcine cysticercosis	Modified Faust’s technique	No eggs of *Taenia* spp. (*N* = 400)	1989	[48]
Mexico	Tropical, arid and semi-arid climate	Soil: in and around houses	Centrifugation/flotation	6% (*N* = 15) (*T. solium*)	1991	[105]
Mexico	Tropical, arid and semi-arid climate	Drinking water	Light microscopy of sediment after centrifugation	8% (*N* = 12) (*T. solium*)	1991	[105]
Mexico	Tropical, arid and semi-arid climate	Objects: Houses of tapeworm carriers (*T. solium*)	Method of Graham: microscopy after collecting eggs with cellulose tape	0% (*N* = 35)	1991	[105]
Mexico	Tropical, arid and semi-arid climate	Soil: In and around houses (toilet, backyard, kitchen, washboard, water containers, corrals)	Centrifugation/flotation	43% of samples positive for *Taenia* spp. eggs in spring (*N* = 109), 7.8% in summer (*N* = 116), 29.2% in autumn (*N* = 113) and 17% in winter (*N* = 53). Highest prevalence in kitchen soil samples	2008	[85]
Nigeria	Tropical savanna climate with hot semi-arid climate in the North	Soil: Playgrounds	Sieving, sedimentation, flotation	36.9% (*N* = 608) (*Taenia* spp./*Echinococcus* spp.) Higher prevalence in dry period	2008	[106]
Peru	Hot desert, tundra and tropical rainforest climate	Soil: Village	Sugar–Percoll sedimentation	2.9% (*N* = 336) (*Taenia* spp.)	2018	[108]
Slovakia	Humid continental climate	Soil: Sandpits	Sheather’s flotation technique	0.7% (*N* = 285) (*Taenia* spp.)	2014	[18]
Turkey	Mixed cold semi-arid, Mediterranean and Continental hot summer climate	Soil: Playgrounds	Zinc sulphate flotation	1% (*N* = 480) (*Taenia* spp.)	2006	[107]
Zimbabwe	Hot semi-arid and dry-winter subtropical highland climate	Drinking water: Boreholes, bowsers, lakes, rivers, springs, taps and wells	Filtration/ centrifugation	*Taenia* spp. eggs found in rivers and lakes (sample size and prevalence not indicated)	2011	[16]

^a^Köppen climate classification

Invertebrates are considered to be possible vectors for the spread of parasitic eggs. In Thailand, one of 820 cockroaches collected in open-air shopping markets in Thailand carried a *Taenia* spp. egg [46], while in Peru, out of 54 pools of 309 wild-caught *Aphodius* spp. beetles, two were positive for *T. solium*, three were positive for *T. hydatigena* and two were positive for other taeniid eggs [47]. In two studies carried out in Mexico, on the other hand, none of the 600 [48) and 1187 [49] flies caught in kitchens carried *Taenia* spp. eggs in their gut.

To confirm the possibility that an invertebrate species might carry and disseminate eggs in the environment, eggs have been fed to selected species in laboratory experiments. Beetles (*Pterostichus vulgaris*, *Aphodius fimetarius*,* A. luridus*,* Ammophorus rubripes*), flies (*Calliphora quadromaculata*, *C. hortona*, *C. stygia*) and earthworms (*Eisenia foulida*,* Lumbricus terrestris* and *Allolobophora caliginosa*) fed with *Taenia* spp. eggs were found to contain eggs in the digestive tract after dissection [19, 50–52]. When beetles (*Ammophorus rubripes*) and blowflies (*Hybopygia varia*,* Calliphora quadromaculata*,* C. hortona* and *C. stygia*) infected in the laboratory with *Taenia* spp. eggs were fed to pigs and lambs, respectively, 94.4% of pigs presented with cysticercosis and all blowflies had transferred the infection [19, 53].

Lawson and Gemmell [19, 20, 54–56] performed several experiments to determine the possible infection route* via* invertebrates and dispersal in the field. Lambs that were allowed to graze downwind of dog kennels or in close proximity to a plot where infected dogs had been previously kept contained a much higher level of cysticerci, detected during autopsy, than those grazing elsewhere. Dead blowflies containing eggs of *T. hydatigena* spread on a pasture were able to transmit infection if ingested by lambs (70% of 14 lambs infected). In another experiment, blowflies were first exposed to *T. pisiformis* eggs by contact with faeces from infected dogs and then afterwards given access to pasture. Five of eight rabbits subsequently allowed to graze on this pasture became infected. In a similar experiment, blowflies were allowed to come into contact with dog faeces contaminated with *T. hydatigena* eggs before they had access to meat. This meat was subsequently fed to pigs, and 100% of the pigs became infected. On the other hand, in experiments where human faeces containing *T. saginata* eggs were deposited 1.5 m from a pasture where calves were grazing, none of the calves contained cysticerci after 8 to 10 weeks [57]. On the Scottish island of St. Kilda, sheep were found to be commonly infected with *T. hydatigena* despite the absence of definitive hosts for this species. Torgerson et al. [58, 59] concluded that eggs had been transported by insects or birds from the nearest inhabited land mass 60 km further away. Lawson and Gemmell [19] also investigated the role of wind in the dispersal of eggs. Faecal samples contaminated with *T. pisiformis* eggs were placed in front of a fan and trays were placed to capture whatever was moved by the draft. The sediment was fed to rabbits, but none became infected.

Evidence for transmission between intermediate hosts does exist. In one experiment, pigs fed with proglottids of *T. solium* were placed among naïve pigs [60]. In each of the four trials, at least one of the naïve pigs became infected, but with much lower cyst intensities compared to the primarily infected pigs. Whether secondary infection was attributable to coprophagic habits is yet to be demonstrated.

### Sewage treatment and surface water

A number of authors have linked access to surface water with a higher risk for cysticercosis, suggesting that eggs either end up in the surface water directly or as they pass through water treatment systems. Kyvsgaard et al. [61] found that allowing cattle access to drink from streams in Denmark was a major risk factor for bovine cysticercosis. Boone et al. [62] reported that the flooding of pastures, free access of cattle to surface water and proximity of wastewater effluent were explanatory variables for bovine cysticercosis in Belgium. In Brazil, the water source from rivers or streams was determined to be the main risk factor for bovine cysticercosis in multiple farms [63]. The flooding of agricultural land and grassland has also been associated with human and porcine cysticercosis in Kenya [64].

Several studies have shown that wastewater treatment plants are not fully capable of removing helminth eggs, including those of *Taenia* spp., from water (Table 4).

**Table 4:** Overview of results on *Taenia* spp. egg presence in the influent/effluent of wastewater treatment systems.

Country	Wastewater treatment system	Egg recovery	Influent	Effluent	Year of publication	Reference
Morocco	Activated sludge and natural lagoon	Modified Bailenger method (sedimentation, centrifugation, flotation and McMaster)	5.28 eggs/l (*N* = 6) and 0 eggs/L (*N* = 6)	0 eggs/l (*N* = 6) and 0 eggs/L (*N* = 6)	2018	[110]
Colombia	Series of anaerobic, facultative and maturation ponds	Modified Bailenger method	63 helminth eggs/l (*N* = 8)	0 eggs/l (*N* = 8)	2002	[111]
Iran	Activated sludge Natural lagoons	Modified Bailenger method	Low number present in 2 of 8 plants (3 and 1.25 eggs/l) (*N* = 16/plant)	< 1 egg/l (*N* = 16/plant)	2006	[112]
Morocco	Stabilization ponds	Bailenger method	0.1 eggs/l (*N* = 48)	0 eggs/l (*N* = 48)	2000	[113]
Tunisia	Activated sludge Stabilization ponds	Bailenger method	From 28 to 208 eggs/l (*N* = 174)	From 17 to 52 eggs/l (*N* = 174)	2009	[88]
Colombia	Anaerobic biodigestors	Filtration, sedimentation, centrifugation and recovery by Sheather and McMaster method	Eggs in 10% of samples (*N* = 80)	Eggs in 10% of samples (*N* = 80)	2012	[114]
South Africa and Lesotho	Centralized plant Decentralized plant	Sieving, sedimentation and flotation	6.4–29.6 eggs/l to 2.3 eggs/l (*N* = 55)	1.4 to 8.4 eggs/l – 0.25 eggs/L (*N* = 55)	2018	[87]
Bolivia	Facultative pond followed by maturation pond	Centrifugation, flotation and biphasic separation	306–3006 eggs/l (*N* = 3)	45 eggs/L (*N* = 3)	2013	[115]
Tunisia	Activated sludge Natural lagoons	Modified Bailenger method	Taeniid eggs in 85% of samples (*N* = 117)	Taeniid eggs in 30% of samples (*N* = 117)	2018	[89]

Newton et al. [65] laboratory tested different treatment processes for their ability to remove *T. saginata* eggs from wastewater. A sedimentation test showed that removal varied from 51 to 98% after 15 and 120 min, respectively. Sand filtration was able to remove 99.6% of eggs from the wastewater and a trickling filter could removed 62–70%.

Eggs that are removed from wastewater in wastewater treatment systems are deposited in the sewage sludge that is formed during the process. Using untreated sludge to fertilize crops and pasture will therefore lead to a higher risk. Several studies have reported that some types of sludge treatment are inadequate in terms of inactivating taeniid eggs (Table 5).

**Table 5:** Overview of results on *Taenia* spp. egg presence in the sludge of wastewater treatment systems

Country	Sludge type and/or treatment	Egg recovery	Presence (*N* = sample size)	Year of publication	Reference
South Africa	Treatment: sludge drying beds for 2 months	Flotation, sedimentation	54 *Taenia* spp. eggs/g with 20 eggs/g viable (*N* = 60)	2018	[90]
Senegal	Treatment: sludge drying beds for 2 months	Flotation, sedimentation	0 eggs (*N* = 3)	2018	[90]
Brazil	Dry matter sludge biosolids	Filtration, sedimentation, centrifugation and flotation	4.85 helminth eggs/g; of which 0.3% *Taenia* spp. (*N* = 22)	1997	[116]
Slovakia	Raw sludge, activated sludge and drained stabilised sludge	Sedimentation, centrifugation, flotation	2.27% raw sludge, 1.14% activated sludge and 2% drained stabilised sludge (*N* = 276)	2015	[117]
France	Anaerobically digested sludge	Modified Faust technique (flotation)	Between 2200 and 2400 *Taenia* spp. eggs/kg* sludge (N = 21)	1990	[118]
England/Wales	/	/	Taeniid eggs in at least one sample/water authority (*N* = 162)	1984	[119]
Morocco	3 systems: Natural lagooning; infiltration–percolation sludge followed by sand filtration; and activated sludge plant	Applied flotation method for the analyses of biowastes	*Taenia* spp. eggs in natural lagooning: 2 eggs/g; in infiltration–percolation sludge: 8 eggs/g; in sand filtration: 2 eggs/g; in activated sludge: 4 eggs/g (*N* not indicated)	2019	[120]
Mexico	Sludge from 3 systems: Conventional APT* with parallel plates; sludge blanket APT; and sand-assisted sedimentation Treatment: Lime stabilization	US EPA technique	90% of helminth eggs destroyed (*N* not indicated)	2000	[91]
Australia	Treatment: Chlorine, copper sulphate, slaked lime, ferric sulphate, UV light, drying, moist heat and cold	*In vivo*: feeding to calves	Only drying, keeping dry for 1 day in the presence of a small amount of common salt and boiling for 5 min killed the eggs (*N* not indicated)	1937	[92]

APT, Advanced primary treatment (coagulation/flocculation/sedimentation); US EPA, United States Environmental Protection Agency

In a study by Ilsøe et al. [66] that was carried out following several outbreaks of bovine cysticercosis in Denmark, illegal application of sludge from septic tanks onto pasture and crops was found to be a frequent source of infection. For livestock permanently housed indoors, the highest risk was fodder as feed (hay harvested from meadow fertilized with septic tank contents and fresh grass harvested after the end of the camping season on camping sites without adequate toilet facilities) or indoor contamination with slurry containing eggs [66]. Newton et al. [65] found that *T. saginata* eggs could survive for months in anaerobic sludge at room temperature; after 200 days, 10–15% of eggs still appeared normal. A study performed by Storey and Phillips [67] showed that eggs of *T. saginata* applied to pasture (in sewage sludge) could still be found on the soil surface after 200 days. Rainfall was able to wash the eggs into the soil where they were protected from radiation and desiccation.

Infectivity of eggs in sludge has been examined using test animals. Olsen and Nansen [68] submerged bags with eggs of *T. taeniaeformis* in sewage sludge of a mesophilic anaerobic batch digester at 35 °C, following which these eggs were placed in mice by an intragastric procedure; cyst recovery declined from 25 to 2% after only 2 days. In Australia, groups of 40 cattle were allowed to graze on pastures irrigated with raw sewage and effluent from lagooning processes, trickling filter plants and activated sludge plants; 30, 3.3, 9 and 12.5% of the animals were found to be infected [69]. In France, however, no cysticerci were found in the heart, masseter muscle, diaphragm and tongue of the cattle that had been allowed to graze on fields to which liquid sewage sludge containing 2.5–4.4 *T. saginata* eggs/g of dry matter had been applied [70]. Control animals that were fed the sludge directly had 1–4 cysticerci in these four body parts. The authors concluded that a 6-week delay between fertilization and grazing was sufficient to inactivate *T. saginata* eggs. A caveat to this study is that low infections are likely to be missed by only dissecting four body parts [70]. In a similar experiment, sheep that were grazing on pasture fertilized with sewage sludge or cattle slurry containing *Taenia* spp. eggs were not infected; however, sheep are not the natural hosts of *T. saginata*, so the result on *Taenia* spp. should be assessed with caution [71].

## Discussion

The results summarized in the review show that as a general rule humidity seems to affect *Taenia* spp. egg survival more than temperature, with low humidity hampering survival (< 34%) [72]. Moderate temperatures (5–25 °C) favour survival [73, 75], while warmer temperatures (> 25°C) shorten survival time [74, 76], as does freezing [77]. Under field conditions, *Taenia* spp. eggs can survive for at least 1 year, as demonstrated by Duthy et al. [28] on Kenyan pastures (*T. saginata*). Other *Taenia* spp. have been shown to survive outdoors for a shorter time period (*T. multiceps*, Wales, [75]), suggesting that survival is dependent on the *Taenia* spp. studied and the outdoor conditions. Since most of the studies included in this review covered only a limited time period and given current knowledge that eggs are able to survive for at least 1 year, the fact that many studies still found eggs to survive at the end of the study period does not allow a solid conclusion to be made on when survival will have decreased to a minimum [73, 76, 78]. The long survival time, certainly under optimal conditions, inevidently increases the chance for an egg to infect a new host and transmit the infection.

The studies retrieved during the literature search mostly describe experiments on egg survival in *Taenia* spp. other than *T. saginata*, *T. asiatica* and *T. solium*. The eggs of these other *Taenia* spp. might be affected in a similar way when put under stress although this is not a certainty; for example, eggs of *Echinococcus granulosus*, which are morphologically identical to those of *Taenia* spp. were still infective after freezing to − 30°C [79].

Several *in vivo* experiments included in this review reported questionable results due to the unknown prior infection status of the experimental animals (e.g. [28]), unknown prior infectivity status of the pasture or the absence of a control for natural infection occurring during the experiment (e.g. [28]). In other experiments, a small sample size was often reported (e.g. [80]). Experiments using *in vivo* techniques, detecting cysticerci in test animals, may be biased because the establishment of cysticerci is highly variable among individual animals [81]. Coman and Rickard [26] found that *in vitro* techniques for assessing the hatching and viability of *T. pisiformis* eggs did not reliably agree with their infectivity in rabbits, indicating that it may not be possible to compare results from studies using *in vitro* and *in vivo* techniques.

There is a lack of recent, structured research on the environmental factors affecting egg survival of the zoonotic *Taenia* spp. Studies on this topic can be complicated by the accessibility of *Taenia* spp. eggs for experimental work. To be able to compare results, homogenous batches of eggs are necessary, but developmental stages and egg infectivity are highly variable between individual tapeworms, between proglottids from the same tapeworm and even within one proglottid [82]. In addition, laboratory extraction and preparation processes may affect the viability of eggs. It should also be noted that working with eggs of *T. solium* is highly hazardous. As a proxy for studies on the survival of eggs of zoonotic *Taenia* spp., eggs of non-zoonotic *Taenia* spp. may be used, which are easier to obtain and do not pose a health hazard in the laboratory. However, although eggs of *Taenia* spp. are morphologically undistinguishable, their resistance to environmental conditions may differ. It is important to obtain species-specific data which may help inform dynamic transmission models for the zoonotic* Taenia* spp. An understanding of the distribution of egg survival times under different conditions would help setting-specific parameterization and greatly facilitate modelling.

The prevalence of *Taenia* spp. eggs found on fruits and vegetables is high, ranging from 0.9 to 33% [41, 83]. These studies were mostly conducted in developing countries where environmental contamination is expected to be higher due to inadequate sanitary practices. The risk for infection in these countries is therefore most likely higher than in Europe, although in Europe *Taenia* spp. DNA was found on up to 28% of samples (purchased from fields, greenhouses and wholesalers) in the spring [17]. After industrial washing, the prevalence is greatly reduced, although little information is available on this subject [41, 45, 84]. Overall, there is a risk for infection for the consumer. Industrial washing is performed using active calcium hypochlorite; regular washing with water might not sufficiently reduce the risk.

In soil and water samples, prevalence ranges from 0 to 43% [48, 85]. Studies analysing soil and water samples were performed in a more varied selection of countries. However, similar to the literature regarding parasite egg prevalence on fruits and vegetables, these articles generally focussed on parasite eggs other than those of *Taenia* spp.; as such, the information available is limited. It has also been shown that egg recovery from vegetables, fruits and the environment (soil and water) was low [86], which may have resulted in underestimation of the data.

Variable survival and initial parasite loads on fruit and vegetables and in the soil and water might be found in other climate zones that are not represented in our review. Hygienic standards could vary significantly among regions, and results may not be relevant for other regions. Contamination of fruits and vegetables could happen at any stage during the transit from the field (where the crop was fertilized) to the processing. Poor personal hygiene and general unsanitary conditions could lead to post-washing contamination and hence transmission [36].

Although there is a good body of information showing that eggs can spread and even infect animals through invertebrates in experimental settings, it remains unclear how likely and how important these scenarios could be in real-life settings. Only four articles considered the parasite egg load of insects caught in the wild, and prevalence in these studies was low.

An important factor in the spread and survival of parasitic eggs is the wastewater treatment system. As seen from the results shown here, egg removal efficiency is very variable in the different systems used in different countries, and many systems were found to be unable to fully remove *Taenia* spp. eggs from the treatment water [87–89], allowing the eggs to spread over larger distances* via* waterways. As egg survival is determined by humidity, eggs are able to survive in water for a long time. Furthermore, several articles pinpointed access to surface water or the proximity of a wastewater treatment plant as risk factors for cysticercosis [61–63].

The inability to remove *Taenia* spp. eggs from the wastewater may be due to the type of wastewater treatment system and its quality. The variability between systems and between parasite egg load in the influent make it difficult to project these results to other regions and wastewater management systems. The papers also focussed on total parasite egg load and provided only limited information on *Taenia* spp.

Most of the eggs end up in the sewage sludge produced during the processing of wastewater [90–92], and experiments have proven that eggs can remain viable for a long time, retaining their infectivity for hosts and thus potentially leading to outbreaks [66–69]. Therefore, using sludge from wastewater treatment plants to fertilize fields on which crops used for animal fodder and human food are subsequently grown could lead to a very high risk of infection. In the EU, the use of sewage sludge in agriculture on land grazed by cattle is restricted and regulated under Council Directive 86/278/EEC [93]. In general, the Directive states that sludge can be used, albeit under conditions in which harmful effects are prevented to soil, vegetation, animals and humans. Sludge must be treated prior to its application on fields by either injecting or working into the soil. In terms of the risk of *Taenia* spp. eggs, there needs to be a minimum of 3 weeks of no grazing or harvesting of crops after treatment with sludge. As it has been demonstrated that eggs remain viable up to 1 year, this period is clearly too short. Some EU countries, however, have a more stringent national legislation compared to the EU directive (Austria, Belgium, Denmark, France, Germany, Netherlands, Sweden) [94].

## Conclusions

In conclusion, the results of this systematic review show that our knowledge of the survival and transmission of *Taenia* spp. eggs in the environment is limited. Indeed, in terms of factors determining egg survival, the results were often doubtful, and in terms of contamination of food, soil, water and the water and sludge from the sewage treatment process, the information was focussed on specific regions (climate zones) or was not specific for *Taenia* spp. Current results indicate that egg survival at moderate temperatures (5–25°C), combined with other conditions favourable for survival (e.g. RH > 80%), together with the large number of factors facilitating egg dispersal (ineffective sewage treatment, contamination of food, possible dispersal in water and soil and to some extent transmission by invertebrates) are making future control/elimination of *Taenia* spp. challenging. Future studies are necessary to identify applicable and sustainable methods to identify and inactivate parasite eggs in the environment and to reduce the spread thereof. Molecular techniques, such as the use of microsatellite markers, to examine genetic variability at the farm or regional level may help unravel specific knowledge gaps. Understanding the epidemiology and the transmission dynamics of *Taenia* spp., and thus approaching egg survival and the dispersal problem from a different angle, might result in new insights and lead to other, possibly more efficient control options.

## Supplementary Information

**Additional file 1:** The protocol used for this review.

** Additional file 2:** PRISMA checklist.

## Data Availability

Not applicable.

## References

[1] Jansen F, Dorny P, Trevisan C, Dermauw V, Laranjo-González M, Allepuz A (2018). Economic impact of bovine cysticercosis and taeniosis caused by *Taenia saginata* in Belgium. Parasites Vectors..

[2] Laranjo-González M, Devleesschauwer B, Jansen F, Dorny P, Dupuy C, Polack B (2018). Epidemiology and economic impact of bovine cysticercosis and taeniosis caused by *Taenia saginata* in North-Eastern Spain (Catalonia). Parasit Vectors..

[3] World Health Organization. Murrell K, editor; Dorny P, Flisser A, Geerts S, Kyvsgaard NC, McManus D, et al., associate editors. WHO/FAO/OIE guidelines for the surveillance, prevention and control of taeniosis/cysticercosis. Geneva: WHO/FAO/OIE; 2005.

[4] Eom K (2006). What is Asian *Taenia*?. Parasitol Int..

[5] Craig P, Ito A (2007). Intestinal cestodes. Curr Opin Infect Dis..

[6] Garcia HH, Gonzalez AE, Evans CAW, Gilman RH (2003). *Taenia solium* cysticercosis. Lancet.

[7] World Health Organization. Ending the neglect to attain the Sustainable Development Goals—a road map for neglected tropical diseases 2021–2030. Geneva: World Health Organization; 2020.

[8] Gripper LB, Welburn SC (2017). The causal relationship between neurocysticercosis infection and the development of epilepsy—a systematic review. Infect Dis Poverty..

[9] Ndimubanzi PC, Carabin H, Budke CM, Nguyen H, Qian Y-J, Rainwater E (2010). A systematic review of the frequency of neurocyticercosis with a focus on people with epilepsy. PLoS Negl Trop Dis..

[10] Dorny P, Praet N (2007). *Taenia saginata* in Europe. Vet Parasitol..

[11] Laranjo-González M, Devleesschauwer B, Gabriël S, Dorny P, Allepuz A (2016). Epidemiology, impact and control of bovine cysticercosis in Europe: a systematic review. Parasites Vectors..

[12] Uygyr-Bayramicli O, Ak O, Dabak R, Demirhan G, Ozer S (2012). *Taenia saginata* a rare cause of acute cholangitis: a case report. Acta Clin Belg..

[13] Bekraki A, Hanna K (2016). Peritonitis caused by jejunal perforation with *Taenia saginata*: report of a case. J Parasit Dis..

[14] Najih M, Laraqui H, Njoumi N, Mouhafid F, Moujahid M, Ehirchiou A, Zentar A (2016). *Taenia saginata*: an unusual cause of post-appendectomy faecal fistula. Pan Afr Med J..

[15] Arriola CS, Gonzalez AE, Gomez-Puerta LA, Lopez-Urbina MT, Garcia HH, Gilman RH (2014). New insights in cysticercosis transmission. PLoS Negl Trop Dis..

[16] Dalu T, Barson M, Nhiwatiwa T (2011). Impact of intestinal microorganisms and protozoan parasites on drinking water quality in Harare. Zimbabwe. J Water Sanit Hyg Develop..

[17] Federer K, Armua-Fernandez MT, Gori F, Hoby S, Wenker C. Deplazes P. Detection of taeniid (*Taenia* spp., *Echinococcus* spp.) eggs contaminating vegetables and fruits sold in European markets and the risk for metacestode infections in captive primates. Int J Parasitol Parasites Wildl. 2016; 5: 249–53. 10.1016/j.ijppaw.2016.07.002 PMC4987496 27556010

[18] Papajova I, Pipikova J, Papaj J, Cizmar A (2014). Parasitic contamination of urban and rural environments in the Slovak Republic: dog’s excrements as a source. Helminthologia..

[19] Lawson JR, Gemmell MA (1985). The potential role of blowflies in the transmission of taeniid tapeworm eggs. Parasitology.

[20] Lawson JR, Gemmell MA (1990). Transmission of taeniid tapeworm eggs via blowflies to intermediate hosts. Parasitology.

[21] Gabriël S, Dorny P, Mwape KE, Trevisan C, Braae UC, Magnussen P, et al. Control of *Taenia Solium* taeniasis/cysticercosis: The best way forward for sub-Saharan Africa? Acta Trop. 2017; 165: 252–60. 10.1016/j.actatropica.2016.04.010 27140860

[22] Pawlowski Z, Singh G (2002). Basic biology and transmission. * Taenia solium* cysticercosis from basic to clinical science.

[23] Brandt JRA, Sewell MMH (1981). In vitro hatching and activation of *Taenia* taeniaeformis oncospheres. Vet Res Commun..

[24] Silverman PH (1954). Studies on the biology of some tapeworms of the genus Taenia. I. Factors affecting hatching and activation of taeniid ova, and some criteria of their viability. Ann Trop Med Parasitol..

[25] Wang IC, Ma YX, Kuo CH, Fan PC (1997). A comparative study on egg hatching methods and oncosphere viability determination for *Taenia solium* eggs. Int J Parasitol..

[26] Coman BJ, Rickard MD (1977). A comparison of in vitro and in vivo estimates of the viability of *Taenia pisiformis* eggs aged under controlled conditions, and their ability to immunize against a challenge infection. Int J Parasitol..

[27] Moher D, Liberati A, Tetzlaff J, Altman DG, PRISMA Group. Preferred reporting items for systematic reviews and meta-analyses: The PRISMA statement. PLoS Med. 2009;6:e1000097. PMC3090117 21603045

[28] Duthy BL, van Someren VD (1948). The survival of *Taenia saginata* on open pasture. East Afr Agri J..

[29] Araújo JM, Araújo JV, Braga FR, Carvalho RO, Silva AR, Campos AK. Interaction and ovicidal activity of nematophagous fungus *Pochonia chlamydosporia* on *Taenia saginata* eggs. Exp Parasitol. 2009; 121: 338–41. 10.1016/j.exppara.2008.12.011 19141298

[30] Araújo JM, Braga FR, Araújo JV, Soares FEF, Geniêr HLA (2010). Biological control of *Taenia saginata* eggs. Helminthologia..

[31] Braga FR, Araújo JV, Carvalho RO, Silva AR, Araújo JM, Tavela AO (2009). Ovicidal effect of nemathophagous fungi on *Taenia taeniaeformis* eggs. World J Microbiol Biotechnol..

[32] Braga FR, Silva AR, Carvalho RO, Araújo JV, Pinto PSA (2011). Ovicidal activity of different concentrations of *Pochonia chlamydosporia* chlamydospores on *Taenia taeniaeformis* eggs. J Helminthol..

[33] Ciarmela ML, Thevenet PS, Alvarez HM, Minvielle MC, Basualdo JA (2005). Effect of *Paecilomyces lilacinus* on the viability of oncospheres of *Taenia hydatigena*. Vet Parasitol..

[34] Prokopič J, Jelenová I (1980). Effect of fertilizers on *Taenia saginata* Goeze, 1782 egg viability in vitro. Folia Parasitol.

[35] Adamu NB, Adamu JY, Mohammed D (2012). Prevalence of helminth parasites found on vegetables sold in Maiduguri. Northeastern Nigeria. Food Control..

[36] Adenusi AA, Abimbola WA, Adewoga TOS (2015). Human intestinal helminth contamination in pre-washed, fresh vegetables for sale in major markets in Ogun state, southwest Nigeria. Food Control.

[37] Fallah AA, Makhtumi Y, Pirali-Kheirabadi K (2016). Seasonal study of parasitic contamination in fresh salad vegetables marketed in Shahrekord. Iran. Food Control..

[38] Ismail Y (2016). Prevalence of parasitic contamination in salad vegetables collected from supermarkets and street vendors in Amman and Baqa’a-Jordan. Pol J Microbiol..

[39] Khan W, Mumtaz G, Bibi S, Afzal S (2017). Parasitic contamination of fresh vegetables sold at upper and lower Dir districts, Khyber Pakhtunkhwa, Pakistan. Pak J Zool..

[40] Maikai BV, Elisha IA, Baba-Onoja EBT (2012). Contamination of vegetables sold in markets with helminth eggs in Zaria metropolis, Kaduna state. Nigeria. Food Control..

[41] Rostami A, Ebrahimi M, Mehravar S, Omrani VF, Fallahi S, Behniafar H (2016). Contamination of commonly consumed raw vegetables with soil transmitted helminth eggs in Mazadaran province, northern Iran. Int J Food Microbiol..

[42] Shahnazi M, Jafari-Sabet M (2010). Prevalence of parasitic contamination of raw vegetables in villages of Qazvin province. Iran. Foodborne Pathog Dis..

[43] Uga S, Hoa NTV, Noda S, Moji K, Cong L, Aoki Y (2009). Parasite egg contamination of vegetables from a suburban market in Hanoi. Vietnam. Nep Med Coll J..

[44] Al-Megrin WAI (2010). Prevalence intestinal parasites in leafy vegetables in Riyadh. Saudi Arabia. Int J Trop Med..

[45] Fallah AA, Pirali-Kheirabadi K, Shirvani F, Saei-Dehkordi SS (2012). Prevalence of parasitic contamination in vegetables used for raw consumption in Shahrekord, Iran: influence of season and washing procedure. Food Control.

[46] Chamavit P, Sahaisook T, Niamnuy N (2011). The majority of cockroaches from the Samutprakarn province of Thailand are carriers of parasitic organisms. EXCLI J..

[47] Vargas-Calla A, Gomez-Puerta LA, Pajuelo MJ, Garcia HH, Gonzalez AE (2018). for the cysticercosis working group in Peru. Molecular detection of taeniid eggs in beetles collected in an area endemic for Taenia solium. Am J Trop Med Hyg..

[48] Keilbach NM, de Aluja AS, Sarti-Gutierrez E (1989). A programme to control taeniasis-cysticercosis (*T. solium*): experiences in a Mexican village. Acta Leidensia..

[49] Martinez MJ, De Aluja AS, Gemmell M (2000). Failure to incrimate domestic flies (Diptera: Muscidae) as mechanical vectors of *Taenia* eggs (Cyclophyllidae: Taeniidae) in rural Mexico. J Med Entomol..

[50] Bily S, Sterba J, Dykova I (1978). Results of an artificial feeding of eggs of *Taenia saginata* Goeze, 1782 to various beetle species. Folia Parasitol.

[51] Gomez-Puerta LA, Lopez-Urbina MT, Garcia HH, Gonzalez AE (2014). Longevity and viability of *Taenia solium* eggs in the digestive system of the beetle *Ammophorus rubripes*. Rev Bras Parasitol Vet..

[52] Lonc E (1980). The possible role of the soil fauna in the epizootiology of cysticercosis in cattle. I. Earthworms—the biotic factor in a transmission of Taenia saginata eggs. Angew Parasitol..

[53] Gomez-Puerta LA, Garcia HH, Gonzalez AE (2018). for the cysticercosis working group in Peru. Experimental porcine cysticercosis using infected beetles with Taenia solium eggs. Acta Trop..

[54] Gemmell MA, Macnamara FN (1976). Factors regulating tapeworm populations: estimations of the infection pressure and index of clustering from *Taenia hydatigena* before and after the removal of infected dogs. Res Vet Sci..

[55] Gemmell MA (1976). Factors regulating tapeworm populations: estimations of the build-up and dispersion patterns of eggs after the introduction of dogs infected with *Taenia hydatigena*. Res Vet Sci..

[56] Gemmell MA, Johnstone PD, Boswell CC (1978). Factors regulating tapeworm populations: dispersion patterns of *Taenia hydatigena* eggs on pasture. Res Vet Sci..

[57] Kyvsgaard NC, Ilsoe B, Henriksen SA, Nansen P (1988). An attempt to evaluate the spreading of *Taenia saginata* eggs in the environment. Acta Vet Scand..

[58] Torgerson PR, Gulland FMD, Gemmell MA (1992). Observations on the epidemiology of *Taenia hydatigena* in Soay sheep on St Kilda. Vet Rec..

[59] Torgerson PR, Pilkington J, Gulland FMD, Gemmell MA (1995). Further evidence for the long-distance dispersal of Taeniid eggs. Int J Parasitol..

[60] Gonzalez AE, LopezUrbina T, Tsang BY, Gavidia CM, Garcia HH (2005). the cysticercosis working group Peru. Short report: secondary transmission in porcine cysticersosis: description and their potential implications for control sustainability. Am J Trop Hyg..

[61] Kyvsgaard NC, Ilsoe B, Willeberg P, Nansen P, Henriksen SA (1991). A case-control study of risk factors in light of *Taenia saginata* cysticercosis in Danish cattle. Acta Vet Scand..

[62] Boone I, Thys E, Marcotty T, de Borchgrave J, Ducheyne E, Dorny P (2007). Distribution and risk factors of bovine cysticercosis in Belgian dairy and mixed herds. Prev Vet Med..

[63] Duarte CTD, Pinto PSA, Silva LF, Santos TO, Bevilacqua PD, Nieto ECA. Epidemiological aspects of cysticercoses in relation to hydrographic net at Triangulo Mineiro, MG, Brazil. Semina: Ciências Agrárias. 2018;39:221–30.

[64] Wardrop NA, Thomas LF, Atkinson PM, de Glanville WA, Cook EAJ (2015). The influence of socio-ecnomic, behavioural and environmental factors on *Taenia spp*. transmission in Western Kenya: evidence from a cross-sectional survey in humans and pigs. PLoS Negl Trop Dis..

[65] Newton WL, Bennett HJ, Figgat WB (1949). Observations on the effect of various sewage treatment processes upon eggs of *Taenia saginata*. Am J Hyg..

[66] Ilsoe B, Kyvsgaard NC, Nansen P, Henriksen SA (1990). Bovine cysticercosis in Denmark: a study of possible causes of infection in farms with heavily infected animals. Acta Vet. Scand..

[67] Storey GW, Philips RA (1985). The survival of parasite eggs throughout the soil profile. Parasitology.

[68] Olsen JE, Nansen P (1990). Infectivity of eggs of *Taenia taeniaeformis* after anaerobic digestion of sewage sludge—a possible model for *Taenia saginata* egg resistance. Acta vet Scand..

[69] Arundel JH, Adolph AJ (1980). Preliminary observations on the removal of *Taenia saginata* eggs from sewage using various treatment processes. Aust Vet J..

[70] Moussavou-Boussougou MN, Geerts S, Madeline M, Ballandonne C, Barbier D, Cabaret J (2005). Sewage sludge or cattle slurry as pasture fertilisers: comparative cysticersosis and trichostrongylosis risk for grazing cattle. Parasitol Res..

[71] Moussavou-Boussougou MN, Dorny P, Cabaret J (2005). Very low helminth infection in sheep grazed on pastures fertilised by sewage sludge or cattle slurry. Vet Parasitol..

[72] Laws GF (1968). Physical factors influencing survival of taeniid eggs. Exp Parasitol..

[73] Froyd G (1962). Longevity of *Taenia saginata* eggs. J Parasitol..

[74] Gemmell MA (1977). Taeniidae: Modification to the life span or the egg and the regulation of tapeworm populations. Exp Parasitol..

[75] Willis JM, Herbert IV (1984). Some factors affecting the eggs of *Taenia multiceps*: their transmission onto pasture and their viability. Ann Trop Med Parasit..

[76] Coman BJ (1975). The survival of *Taenia pisiformis* eggs under laboratory conditions and in the field environment. Aust Vet J..

[77] Lucker JT (1960). A test of resistance of *Taenia saginata* eggs to freezing. J Parasitol..

[78] Bucur I, Gabriël S, Van Damme I, Dorny P, Johansen MV (2019). Survival of *Taenia saginata* eggs under different environmental conditions. Vet Parasitol..

[79] Colli CW, Williams JF (1972). Influence of temperature on the infectivity of eggs of *Echinococcus granulosus* in laboratory rodents. J Parasitol..

[80] Ilsoe B, Kyvsgaard NC, Nansen P, Henriksen SA (1990). A study on the survival of *Taenia saginata* eggs on soil in Denmark. Acta Vet Scand..

[81] Deckers N, Kanobana K, Silva M, Gonzalez AE, Garcia HH, Gilman RH (2008). Serological responses in porcine cysticercosis: A link with the parasitological outcome of infection. Int J Parasitol..

[82] Geerts S (1990). *Taenia saginata*: an eternal problem?. Verh K Acad Geneeskd Belg..

[83] Abougrain AK, Nahaisi MH, Madi NS, Saied MM, Ghenghesh KS (2010). Parasitological contamination in salad vegetables in Tripoli, Libya. Food Control.

[84] Kozan E, Gonenc B, Sarimehmetoglu O, Aycicek H (2005). Prevalence of helminth eggs on raw vegetables used for salads. Food Control.

[85] Huerta M, Avila R, Jiménez HI, Diaz R, Diaz Huerta ME, Hernandez M (2008). Parasite contamination of soil in households of a Mexican rural community endemic for neurocysticersosis. Trans R Soc Trop Med Hyg..

[86] Scandrett WB, Gajadhar AA (2004). Recovery of putative taeniid eggs from silt in water associated with an outbreak of bovine cysticercosis. Can. Vet. J..

[87] Amoah ID, Reddy P, Seidu R, Stenstrom TA (2018). Removal of helminth eggs by centralized and decentralized wastewater treatment plants in South Africa and Lesotho: health implications for direct and indirect exposure to the effluents. Environ Sci Pollut Res..

[88] Ben Ayed L, Schijven J, Alouini Z, Jemli M, Sabbahi S (2009). Presence of parasitic protozoa and helminth in sewage and efficiency of sewage treatment in Tunisia. Parasitol Res..

[89] Sabbahi S, Trad M, Ben Ayed L, Marzougui N (2018). Occurrence of intestinal parasites in sewage samples and efficiency of wastewater treatment systems in Tunisia. Water Qual Res J..

[90] Amoah ID, Reddy P, Seidu R, Stenstrom TA (2018). Concentration of soil-transmitted helminth eggs in sludge from South-Africa and Senegal: a probabilistic estimation of infection risks associated with agricultural application. J Environ Manage..

[91] Jimenez B, Barrios JA, Maya C (2000). Class B biosolids production from wastewater sludge with high pathogenic content generated in an advanced primary treatment. Water Sci Technol..

[92] Penfold WJ, Penfold HB, Phillips M (1937). The criteria of life and viability of mature *Taenia saginata* ova. Med J Aust..

[93] European Economic Community. Council Directive 86/278/EEC of 12 June 1986 on the protection of the environment, and in particular of the soil, when sewage sludge is used in agriculture. Off J Euro Community. 1986;4:6e12.

[94] European Commission. Disposal and recycling routes for sewage sludge. Part 2—regulatory report. Off J Euro Community. 2001. http://ec.europa.eu/environment/archives/waste/sludge/pdf/sludge_disposal2.pdf. Accessed 20 May 2020

[95] Buttar BS, Nelson ML, Busboom JR, Hancock DD, Walsh DB, Jasmer DP (2013). Effect of heat treatment on viability of Taenia hydatigena eggs. Exp Parasitol.

[96] Thevenet PS, Alvarez HM, Basualdo JA (2017). Survival, physical and physiological changes of *Taenia hydatigena* eggs under different conditions of water stress. Exp Parasitol..

[97] Maya C, Ortiz M, Jiménez B (2010). Viability of *Ascaris* and other helminth genera non larval eggs in different conditions of temperature, lime (pH) and humidity. Water Sci Technol..

[98] Buttar BS, Nelson ML, Busboom JR, Hancock DD, Walsh DB, Jasmer DP (2013). Effect of ensilation of patato on viability of *Taenia hydatigena* eggs. Exp Parasitol..

[99] Negita T, Ito A (1994). In vitro hatching of oncospheres of *Taenia taeniaeformis* using eggs isolated from fresh, frozen, formalin-fixed and ethanol-fixed segments. J Helminthol..

[100] Konno K, Oku Y, Sakai H, Kamiya M (1997). Effect of ultraviolet radiation on the infectivity of *Taenia taeniaeformis* eggs. Jpn J Vet Res..

[101] Lagapa JTG, Konno K, Oku Y, Nonaka N, Kamiya M (2001). Inhibitory effect of different UV lamps on the infectivity of taeniid eggs. Parasitol Res..

[102] Maya C, Torner-Morales FJ, Lucario ES, Hernández E, Jiménez B (2012). Viability of six species of larval and non-larval helminth eggs for different conditions of temperature, pH and dryness. Water Res..

[103] Adanir R, Tasci F (2013). Prevalence of helminth eggs in raw vegetables consumed in Burdur. Turkey. Food Control..

[104] Nooraldeen K (2015). Contamination of public squares and parks with parasites in Erbil city. Irak. Ann Agri Environ Med..

[105] Camacho SPD, Ruiz AC, Peraza VS, Ramos MLZ, Medina MF, Lozano R (1991). Epidemiologic study and control of *Taenia solium* infections with praziquantel in a rural village of Mexico. Am J Trop Med Hyg..

[106] Maikai BV, Umoh JU, Ajanusi OJ, Ajogi I (2008). Public health implications of soil contaminated with helminth eggs in the metropolis of Kadanu. Nigeria. J Helminthol..

[107] Özkayhan MA (2006). Soil contamination with ascarid eggs in playground in Kirikkale. Turkey. J Helminthol..

[108] Pray IW, Gamboa R, Elizalde M, Gomez LA, Vilchez P, Muro C (2018). Detection of *Taenia* eggs in soil after mass anti-helminthic treatment: results from a community-wide soil sampling in Northern Peru. Am Soc Trop Med Hyg..

[109] Aghaindum AG, Landry FKA (2019). Dissemination of the resistant forms of intestinal worms in the marshy areas of the city of Yaounde (Cameroon): importance of some abiotic factors of the medium. Appl Water Sci..

[110] Chaoua S, Boussaa S, Khadra A, Boumezzough A (2018). Efficiency of two sewage treatment systems (activated sludge and natural lagoons) for helminth egg removal in Morocco. J Infect Public Health.

[111] Madera CA, Pena MR, Mara DD (2002). Microbiological quality of waste stabilization pond effluent used for restricted irrigation in Valle Del Cauca. Colombia. Water Sci Technol..

[112] Mahvi AH, Kia EB (2006). Helminth eggs in raw and treated wastewater in the Islamic Republic of Iran. East Mediter Health J..

[113] Bouhoum K, Amahmid O, Asmama S (2000). Occurrence and removal of protozoan cysts and helminth eggs in waste stabilization ponds in Marrakech. Water Sci Technol..

[114] Canon-Franco WA, Henao-Agudelo RA, Perez-Bedoya JL (2012). Recovery of gastrointestinal swine parasites in anaerobic biodigester systems. Rev Bras Parasitol Vet..

[115] Verbyla ME, Oakley SM, Lizima LA, Zhang J, Iriarte M, Tejada-Martinez AE (2013). Taenia eggs in a stabilization pond system with poor hydraulics: concern for human cysticersosis?. Water Sci Technol..

[116] Thomaz-Soccol V, Paulino RC, Castro EA, Andreoli CV (1997). Helminth eggs viability in sewage and biosolids sludge in Curitiba, Parana. Braz Arquivos Biol Technol..

[117] Dudlova A, Juris P, Jarcuska P, Cislakova L, Papajova I, Krcmery V (2015). Epidemiological risks of endoparasitoses spread by municipal wastewater. Helmintologia..

[118] Barbier D, Perrine D, Duhamel C, Doublet R, Georges P (1990). Parasitic hazard with sewage sludge applied to land. Appl Environ Microbiol..

[119] Crewe W (1984). The transmission of *Taenia saginata* in Britain. Ann Trop Med Parasitol..

[120] El Fels L, El Hayany B, El Faiz A, Saadani M, Houari M, Hafidi M (2019). Sludge nematodes, cestodes, activated sludge and infiltration–percolation wastewater treatment system under semi-arid climate. Env Sci Poll Res..

