# Supplementary material for: The survival and dispersal of Taenia eggs in the environment: what are the implications for transmission? A systematic review
Source: Parasit Vectors. 2021 Jan 29;14:88. doi: 10.1186/s13071-021-04589-6 (PMC7845101; doi:10.1186/s13071-021-04589-6)
Supplement: Supplementary file 1 — Additional file 1: The protocol used for this review. [file 13071_2021_4589_MOESM1_ESM.docx]

**Additional file 1**

Title: ***Taenia* spp. eggs in the environment: what are the implications for transmission? – a systematic review**

Authors: Famke Jansen, Sarah Gabriël, Veronique Dermauw, Pierre Dorny, Maria Vang Johansen, Chiara Trevisan

Research question

**Which factors influence the survival and dispersal of *Taenia* spp. eggs in the environment? And How do these impact on transmission?**

**Environment includes: water, sewage, soil, feed and animals (e.g. insects, birds etc.)**

**Databases**

PubMed; Web of Science

**Years:** no restriction on start date - 31 July 2019

**Geographical restriction**: none

**Language:** English

Search phrase

taeni* AND egg* AND (surviv* OR viab* OR resist* OR longevi* OR activ* OR hatch* OR transmi* OR epi* OR infectiv* OR water OR wastewater OR sewage OR sludge OR river OR stream OR soil OR silt OR grass OR saline OR environment* OR medi*)

**Exclusion criteria (Title and Abstract)**

- Reviews
- Editorial letters

**Exclusion criteria (full text)**

- Full text not available
- Non T*aenia* spp.
- Outside the scope of the review (egg survival and dispersal) (so no lab techniques for hatching)

**Database (Excel file)**

- Excel database for screening and data extraction

**Results**

PRISMA 2009 Flow Diagram

Descriptive - Summarizing survival and dispersal conditions of the eggs
